# 2,6-Bis[(*S*)-1-phenyl­eth­yl]-1*H*,5*H*-pyrrolo­[3,4-*f*]isoindole-1,3,5,7(2*H*,6*H*)-tetrone

**DOI:** 10.1107/S1600536812007684

**Published:** 2012-02-29

**Authors:** Alaa A.-M. Abdel-Aziz, Adel S. El-Azab, Amer M. Alanazi, Seik Weng Ng, Edward R. T. Tiekink

**Affiliations:** a Department of Pharmaceutical Chemistry, College of Pharmacy, King Saud University, Riyadh 11451, Saudi Arabia; b Department of Medicinal Chemistry, Faculty of Pharmacy, University of Mansoura, Mansoura 35516, Egypt; c Department of Organic Chemistry, Faculty of Pharmacy, Al-Azhar University, Cairo 11884, Egypt; d Department of Chemistry, University of Malaya, 50603 Kuala Lumpur, Malaysia; e Chemistry Department, Faculty of Science, King Abdulaziz University, PO Box 80203 Jeddah, Saudi Arabia

## Abstract

In the title compound, C_26_H_20_N_2_O_4_, the central isoindole core is almost planar (r.m.s. deviation = 0.043 Å). The phenyl rings lie to either side of the plane [dihedral angles = 88.64 (5) and 67.74 (6)°] and the dihedral angle between the phenyl rings is 63.39 (7)°. In the crystal, mol­ecules are linked by C—H⋯O inter­actions; notably, one carbonyl O atom accepts three such bonds.

## Related literature

For the biological activity of cyclic imides including that of the title compound, see: Abdel-Aziz (2007 ▶); Abdel-Aziz, El-Azab *et al.* (2011 ▶); Abdel-Aziz, ElTahir *et al.* (2011 ▶).
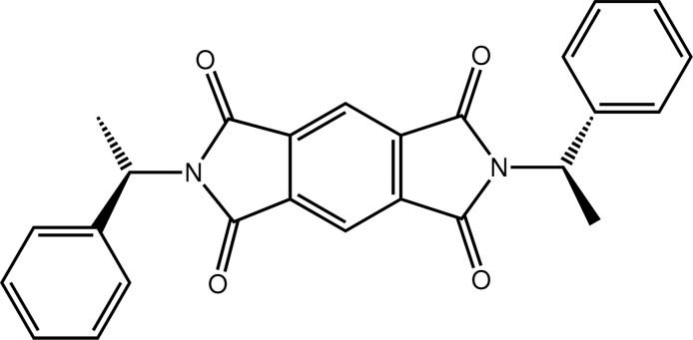


## Experimental

### 

#### Crystal data

C_26_H_20_N_2_O_4_
*M* *_r_* = 424.44
Monoclinic, 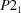

*a* = 5.6401 (1) Å
*b* = 16.1040 (2) Å
*c* = 11.3759 (2) Å
β = 99.762 (2)°
*V* = 1018.29 (3) Å^3^
*Z* = 2
Cu *K*α radiation
μ = 0.77 mm^−1^
*T* = 100 K
0.30 × 0.25 × 0.20 mm

#### Data collection

Agilent SuperNova Dual diffractometer with an Atlas detector
Absorption correction: multi-scan (*CrysAlis PRO*; Agilent, 2011 ▶) *T*_min_ = 0.656, *T*_max_ = 1.000
7858 measured reflections
4093 independent reflections
4091 reflections with *I* > 2σ(*I*)
*R*_int_ = 0.018

#### Refinement

*R*[*F* ^2^ > 2σ(*F* ^2^)] = 0.033
*wR*(*F* ^2^) = 0.099
*S* = 1.11
4093 reflections
289 parameters
1 restraint
H-atom parameters constrained
Δρ_max_ = 0.24 e Å^−3^
Δρ_min_ = −0.25 e Å^−3^
Absolute structure: Flack (1983 ▶), 1898 Friedel pairs
Flack parameter: 0.08 (12)

### 

Data collection: *CrysAlis PRO* (Agilent, 2011 ▶); cell refinement: *CrysAlis PRO*; data reduction: *CrysAlis PRO* ; program(s) used to solve structure: *SHELXS97* (Sheldrick, 2008 ▶); program(s) used to refine structure: *SHELXL97* (Sheldrick, 2008 ▶); molecular graphics: *ORTEP-3* (Farrugia, 1997 ▶) and *DIAMOND* (Brandenburg, 2006 ▶); software used to prepare material for publication: *publCIF* (Westrip, 2010 ▶).

## Supplementary Material

Crystal structure: contains datablock(s) global, I. DOI: 10.1107/S1600536812007684/bt5823sup1.cif

Structure factors: contains datablock(s) I. DOI: 10.1107/S1600536812007684/bt5823Isup2.hkl

Supplementary material file. DOI: 10.1107/S1600536812007684/bt5823Isup3.cml

Additional supplementary materials:  crystallographic information; 3D view; checkCIF report

## Figures and Tables

**Table 1:** Hydrogen-bond geometry (Å, °)

*D*—H⋯*A*	*D*—H	H⋯*A*	*D*⋯*A*	*D*—H⋯*A*
C6—H6⋯O1^i^	0.95	2.50	3.4129 (16)	162
C7—H7⋯O4^ii^	1.00	2.49	3.1840 (16)	126
C20—H20*C*⋯O1^iii^	0.98	2.56	3.4948 (17)	161
C23—H23⋯O1^iv^	0.95	2.57	3.4847 (18)	161

Symmetry codes: (i) 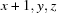; (ii) 
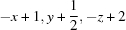
; (iii) 
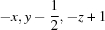
; (iv) 
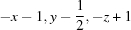
.

**Fig. 1.**
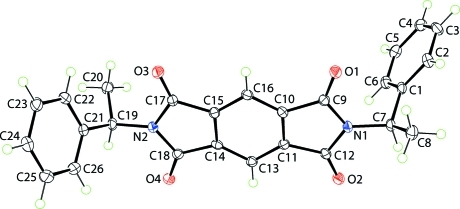
The molecular structure of (I) showing the atom-labelling scheme and displacement ellipsoids at the 50% probability level.

**Fig. 2.**
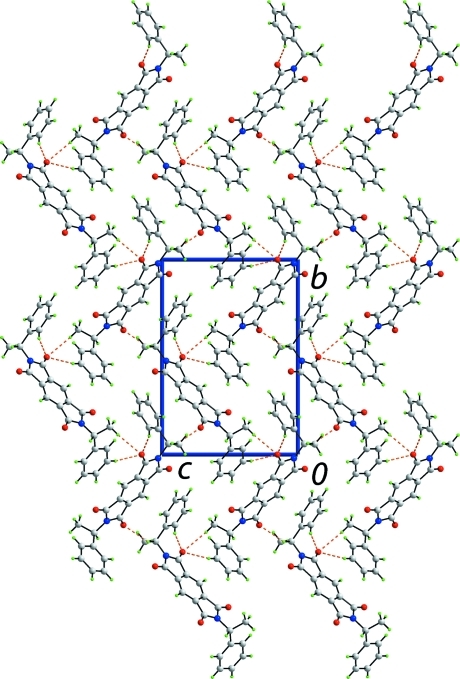
A view in projection down the a axis of the unit-cell contents for (I). The C—H···O interactions are shown as orange dashed lines.

## supplementary crystallographic information

### Comment

Recent work has shown that cyclic imides possess important biological potential, such as anti-hyperlipidemic, anti-diabetic, anti-tumour and anti-inflammatory activities (Abdel-Aziz, 2007; Abdel-Aziz, El-Azab *et al.*, 2011; Abdel-Aziz, ElTahir *et al.*, 2011). The title cyclic imide, 2,6-bis((*S*)-1-phenylethyl)pyrrolo[3,4-*f*]isoindole-1,3,5,7(2*H*,6*H*)-tetraone (I), was investigated during these trials and herein the crystal structure determination is described.

In (I), Fig. 1, the 12 atoms comprising the isoindole core are co-planar with a r.m.s. deviation for the fitted atoms of 0.043 Å. The maximum deviations from this least-squares plane are 0.043 (1) Å for the C15 and C16 atoms, and -0.058 (1) Å for the N2 atom. The phenyl rings lie to either side of the plane, forming dihedral angles of 88.64 (5)° [C1–C6] and 67.74 (6)° [C21–C26]; the dihedral angle between the phenyl rings = 63.39 (7)°.

Molecules are assembled in the crystal structure by C–H···O interactions, Fig. 2 and Table 1. Notably, one carbonyl-O atom is involved in a trifurcated H bond.

### Experimental

A solution of (+)-(*S*)-1-phenylethanamine (10 mmol) and benzene-1,2,4,5-tetracarboxylic dianhydride (10 mmol) in glacial acetic acid (10 ml) was heated under reflux for 12 h. After the evaporation of the reaction mixture to dryness under reduced pressure, the residue was neutralized using sodium bicarbonate solution (4%) until effervescence ceased. The precipitate obtained was washed with water, dried and recrystallized from MeOH/CHCl_3_ (1:1 *v*/*v*). Yield 59%. *M*.pt: > 623 K. ^1^H NMR (CDCl_3_): δ 8.20 (s, 2H, Ar—H), 7.53–7.51 (d, 4H, J = 7.0 Hz, Ar—H), 7.38–7.35 (t, 4H, J = 7.0 Hz, Ar—H), 7.32–7.28 (q, 2H, J = 8.0 Hz, Ar—H), 5.62–5.61 (d, 2H, J = 6.0 Hz, 2CH), 1.98–1.96 (d, 6H, J = 6.0 Hz, 2CH_3_). ^13^C NMR (CDCl_3_): δ 166.02, 139.52, 137.05, 128.64, 128.07, 127.49, 118.19, 50.52, 17.41.

### Refinement

Carbon-bound H-atoms were placed in calculated positions [C—H = 0.95 to 1.00 Å, *U*_iso_(H) = 1.2–1.5*U*_eq_(C)] and were included in the refinement in the riding model approximation. The absolute configuration was determined from 1898 Friedel pairs, and was that of the starting reactant.

### Crystal data

**Table:**

C_26_H_20_N_2_O_4_	*F*(000) = 444
*M**_r_* = 424.44	*D*_x_ = 1.384 Mg m^−^^3^
Monoclinic, *P*2_1_	Cu *K*α radiation, λ = 1.5418 Å
Hall symbol: P 2yb	Cell parameters from 6911 reflections
*a* = 5.6401 (1) Å	θ = 2.7–76.5°
*b* = 16.1040 (2) Å	µ = 0.77 mm^−^^1^
*c* = 11.3759 (2) Å	*T* = 100 K
β = 99.762 (2)°	Prism, colourless
*V* = 1018.29 (3) Å^3^	0.30 × 0.25 × 0.20 mm
*Z* = 2	

### Data collection

**Table:**

Agilent SuperNova Dual diffractometer with an Atlas detector	4093 independent reflections
Radiation source: SuperNova (Cu) X-ray Source	4091 reflections with *I* > 2σ(*I*)
Mirror monochromator	*R*_int_ = 0.018
Detector resolution: 10.4041 pixels mm^-1^	θ_max_ = 76.7°, θ_min_ = 3.9°
ω scan	*h* = −7→7
Absorption correction: multi-scan (*CrysAlis PRO*; Agilent, 2011)	*k* = −20→20
*T*_min_ = 0.656, *T*_max_ = 1.000	*l* = −14→13
7858 measured reflections	

### Refinement

**Table:**

Refinement on *F*^2^	Secondary atom site location: difference Fourier map
Least-squares matrix: full	Hydrogen site location: inferred from neighbouring sites
*R*[*F*^2^ > 2σ(*F*^2^)] = 0.033	H-atom parameters constrained
*wR*(*F*^2^) = 0.099	*w* = 1/[σ^2^(*F*_o_^2^) + (0.0781*P*)^2^ + 0.0806*P*] where *P* = (*F*_o_^2^ + 2*F*_c_^2^)/3
*S* = 1.11	(Δ/σ)_max_ < 0.001
4093 reflections	Δρ_max_ = 0.24 e Å^−^^3^
289 parameters	Δρ_min_ = −0.25 e Å^−^^3^
1 restraint	Absolute structure: Flack (1983), 1898 Friedel pairs
Primary atom site location: structure-invariant direct methods	Flack parameter: 0.08 (12)

### Special details

**Table:**

Geometry. All s.u.'s (except the s.u. in the dihedral angle between two l.s. planes) are estimated using the full covariance matrix. The cell s.u.'s are taken into account individually in the estimation of s.u.'s in distances, angles and torsion angles; correlations between s.u.'s in cell parameters are only used when they are defined by crystal symmetry. An approximate (isotropic) treatment of cell s.u.'s is used for estimating s.u.'s involving l.s. planes.
Refinement. Refinement of *F*^2^ against ALL reflections. The weighted *R*-factor *wR* and goodness of fit *S* are based on *F*^2^, conventional *R*-factors *R* are based on *F*, with *F* set to zero for negative *F*^2^. The threshold expression of *F*^2^ > 2σ(*F*^2^) is used only for calculating *R*-factors(gt) *etc*. and is not relevant to the choice of reflections for refinement. *R*-factors based on *F*^2^ are statistically about twice as large as those based on *F*, and *R*- factors based on ALL data will be even larger.

### Fractional atomic coordinates and isotropic or equivalent isotropic displacement parameters (Å^2^)

**Table:**

	*x*	*y*	*z*	*U*_iso_*/*U*_eq_	
O1	−0.21322 (17)	0.49962 (6)	0.84958 (9)	0.0190 (2)	
O2	0.52962 (18)	0.42601 (6)	1.05125 (9)	0.0204 (2)	
O3	−0.18870 (17)	0.21623 (6)	0.51186 (8)	0.0194 (2)	
O4	0.55058 (17)	0.13790 (6)	0.71548 (9)	0.0198 (2)	
N1	0.1580 (2)	0.48014 (7)	0.97059 (10)	0.0157 (2)	
N2	0.1824 (2)	0.15891 (7)	0.59322 (10)	0.0154 (2)	
C1	0.1785 (2)	0.63194 (8)	0.97086 (11)	0.0156 (3)	
C2	0.0123 (3)	0.69641 (9)	0.96693 (13)	0.0197 (3)	
H2	−0.1151	0.6924	1.0116	0.024*	
C3	0.0320 (3)	0.76653 (9)	0.89804 (14)	0.0224 (3)	
H3	−0.0829	0.8099	0.8954	0.027*	
C4	0.2184 (3)	0.77357 (9)	0.83304 (13)	0.0214 (3)	
H4	0.2327	0.8219	0.7870	0.026*	
C5	0.3837 (2)	0.70929 (10)	0.83595 (12)	0.0212 (3)	
H5	0.5106	0.7133	0.7909	0.025*	
C6	0.3636 (2)	0.63922 (9)	0.90455 (12)	0.0184 (3)	
H6	0.4776	0.5956	0.9063	0.022*	
C7	0.1695 (2)	0.55439 (8)	1.04732 (11)	0.0158 (3)	
H7	0.3270	0.5517	1.1029	0.019*	
C8	−0.0248 (3)	0.55292 (10)	1.12469 (13)	0.0211 (3)	
H8A	−0.0150	0.5007	1.1696	0.032*	
H8B	−0.1831	0.5573	1.0740	0.032*	
H8C	−0.0017	0.5998	1.1804	0.032*	
C9	−0.0287 (2)	0.46074 (8)	0.87821 (11)	0.0153 (3)	
C10	0.0478 (2)	0.38325 (8)	0.82149 (12)	0.0146 (3)	
C11	0.2743 (2)	0.36088 (8)	0.88183 (11)	0.0142 (3)	
C12	0.3478 (2)	0.42320 (8)	0.97899 (12)	0.0150 (3)	
C13	0.4002 (2)	0.29243 (8)	0.85060 (11)	0.0147 (3)	
H13	0.5538	0.2767	0.8927	0.018*	
C14	0.2814 (2)	0.24907 (8)	0.75224 (12)	0.0141 (2)	
C15	0.0553 (2)	0.27140 (8)	0.69167 (11)	0.0144 (3)	
C16	−0.0710 (2)	0.33948 (9)	0.72418 (12)	0.0151 (3)	
H16	−0.2258	0.3547	0.6831	0.018*	
C17	−0.0107 (2)	0.21467 (8)	0.58745 (12)	0.0150 (3)	
C18	0.3646 (2)	0.17566 (8)	0.68996 (11)	0.0146 (2)	
C19	0.2125 (2)	0.09707 (8)	0.50044 (12)	0.0158 (3)	
H19	0.3719	0.0698	0.5268	0.019*	
C20	0.2275 (3)	0.14171 (9)	0.38372 (12)	0.0201 (3)	
H20A	0.3534	0.1843	0.3978	0.030*	
H20B	0.0726	0.1680	0.3533	0.030*	
H20C	0.2662	0.1016	0.3251	0.030*	
C21	0.0229 (2)	0.02911 (8)	0.49308 (12)	0.0164 (3)	
C22	−0.1846 (3)	0.02927 (9)	0.40638 (12)	0.0189 (3)	
H22	−0.2102	0.0727	0.3491	0.023*	
C23	−0.3546 (3)	−0.03401 (9)	0.40349 (13)	0.0207 (3)	
H23	−0.4949	−0.0336	0.3440	0.025*	
C24	−0.3199 (3)	−0.09748 (9)	0.48692 (14)	0.0218 (3)	
H24	−0.4374	−0.1399	0.4857	0.026*	
C25	−0.1120 (3)	−0.09870 (9)	0.57237 (14)	0.0249 (3)	
H25	−0.0862	−0.1425	0.6291	0.030*	
C26	0.0579 (3)	−0.03601 (10)	0.57488 (12)	0.0217 (3)	
H26	0.2000	−0.0375	0.6332	0.026*	

### Atomic displacement parameters (Å^2^)

**Table:**

	*U*^11^	*U*^22^	*U*^33^	*U*^12^	*U*^13^	*U*^23^
O1	0.0176 (4)	0.0184 (5)	0.0201 (5)	0.0040 (4)	0.0006 (3)	−0.0031 (4)
O2	0.0200 (5)	0.0155 (5)	0.0229 (5)	−0.0003 (4)	−0.0038 (4)	−0.0023 (4)
O3	0.0173 (5)	0.0189 (5)	0.0201 (5)	0.0021 (4)	−0.0022 (4)	−0.0031 (4)
O4	0.0201 (5)	0.0184 (5)	0.0195 (4)	0.0061 (4)	−0.0005 (4)	−0.0008 (4)
N1	0.0179 (5)	0.0116 (6)	0.0170 (5)	0.0003 (4)	0.0013 (4)	−0.0026 (4)
N2	0.0164 (5)	0.0135 (5)	0.0156 (5)	0.0013 (4)	0.0009 (4)	−0.0017 (4)
C1	0.0153 (6)	0.0133 (6)	0.0173 (6)	−0.0007 (5)	−0.0001 (4)	−0.0050 (5)
C2	0.0197 (6)	0.0158 (7)	0.0245 (7)	0.0008 (5)	0.0064 (5)	−0.0033 (5)
C3	0.0242 (7)	0.0131 (6)	0.0280 (7)	0.0039 (5)	−0.0011 (6)	−0.0022 (5)
C4	0.0260 (7)	0.0161 (7)	0.0199 (6)	−0.0032 (5)	−0.0022 (5)	−0.0006 (5)
C5	0.0212 (7)	0.0234 (7)	0.0188 (6)	−0.0040 (6)	0.0032 (5)	−0.0036 (5)
C6	0.0162 (6)	0.0180 (7)	0.0207 (6)	0.0017 (5)	0.0023 (5)	−0.0042 (5)
C7	0.0182 (6)	0.0119 (6)	0.0168 (6)	−0.0008 (5)	0.0014 (4)	−0.0041 (5)
C8	0.0243 (7)	0.0191 (6)	0.0208 (6)	−0.0041 (5)	0.0060 (5)	−0.0033 (5)
C9	0.0183 (6)	0.0127 (6)	0.0146 (6)	−0.0019 (5)	0.0023 (5)	0.0004 (5)
C10	0.0161 (6)	0.0115 (6)	0.0163 (6)	0.0001 (5)	0.0030 (5)	0.0006 (5)
C11	0.0154 (6)	0.0125 (6)	0.0141 (6)	−0.0028 (5)	0.0012 (5)	0.0012 (5)
C12	0.0173 (6)	0.0108 (6)	0.0169 (6)	−0.0020 (5)	0.0024 (5)	0.0011 (5)
C13	0.0146 (6)	0.0136 (6)	0.0155 (6)	−0.0009 (5)	0.0015 (4)	0.0013 (5)
C14	0.0159 (6)	0.0112 (6)	0.0155 (5)	−0.0007 (5)	0.0030 (4)	0.0037 (5)
C15	0.0150 (6)	0.0129 (6)	0.0149 (6)	−0.0019 (5)	0.0014 (4)	0.0004 (4)
C16	0.0145 (6)	0.0133 (6)	0.0166 (6)	0.0009 (5)	0.0004 (4)	0.0011 (4)
C17	0.0163 (6)	0.0125 (6)	0.0160 (6)	−0.0002 (5)	0.0025 (4)	−0.0013 (5)
C18	0.0156 (6)	0.0143 (6)	0.0139 (6)	−0.0013 (5)	0.0028 (4)	0.0018 (5)
C19	0.0169 (6)	0.0144 (6)	0.0163 (6)	0.0015 (5)	0.0033 (4)	−0.0046 (5)
C20	0.0235 (6)	0.0193 (7)	0.0180 (6)	−0.0019 (5)	0.0045 (5)	−0.0021 (5)
C21	0.0196 (6)	0.0133 (6)	0.0162 (6)	0.0019 (5)	0.0027 (5)	−0.0035 (5)
C22	0.0204 (6)	0.0148 (6)	0.0205 (6)	0.0022 (5)	0.0007 (5)	0.0004 (5)
C23	0.0184 (6)	0.0190 (7)	0.0232 (7)	0.0014 (5)	−0.0011 (5)	−0.0039 (5)
C24	0.0239 (6)	0.0157 (7)	0.0260 (7)	−0.0019 (5)	0.0046 (5)	−0.0024 (5)
C25	0.0316 (7)	0.0196 (7)	0.0223 (7)	−0.0027 (6)	0.0012 (6)	0.0040 (5)
C26	0.0238 (7)	0.0203 (7)	0.0189 (6)	−0.0003 (6)	−0.0024 (5)	0.0005 (5)

### Geometric parameters (Å, º)

**Table:**

O1—C9	1.2111 (17)	C10—C16	1.3855 (19)
O2—C12	1.2017 (16)	C10—C11	1.3915 (18)
O3—C17	1.2062 (16)	C11—C13	1.3897 (19)
O4—C18	1.2049 (16)	C11—C12	1.4984 (18)
N1—C9	1.3911 (16)	C13—C14	1.3909 (18)
N1—C12	1.4004 (17)	C13—H13	0.9500
N1—C7	1.4754 (16)	C14—C15	1.3902 (18)
N2—C18	1.3986 (16)	C14—C18	1.4941 (18)
N2—C17	1.4046 (16)	C15—C16	1.3913 (18)
N2—C19	1.4815 (16)	C15—C17	1.4930 (17)
C1—C6	1.3928 (18)	C16—H16	0.9500
C1—C2	1.3945 (18)	C19—C21	1.5222 (19)
C1—C7	1.5276 (18)	C19—C20	1.5249 (18)
C2—C3	1.390 (2)	C19—H19	1.0000
C2—H2	0.9500	C20—H20A	0.9800
C3—C4	1.389 (2)	C20—H20B	0.9800
C3—H3	0.9500	C20—H20C	0.9800
C4—C5	1.390 (2)	C21—C26	1.394 (2)
C4—H4	0.9500	C21—C22	1.3970 (18)
C5—C6	1.388 (2)	C22—C23	1.396 (2)
C5—H5	0.9500	C22—H22	0.9500
C6—H6	0.9500	C23—C24	1.386 (2)
C7—C8	1.5175 (18)	C23—H23	0.9500
C7—H7	1.0000	C24—C25	1.391 (2)
C8—H8A	0.9800	C24—H24	0.9500
C8—H8B	0.9800	C25—C26	1.389 (2)
C8—H8C	0.9800	C25—H25	0.9500
C9—C10	1.5012 (18)	C26—H26	0.9500
			
C9—N1—C12	112.10 (11)	C11—C13—C14	113.97 (11)
C9—N1—C7	125.51 (11)	C11—C13—H13	123.0
C12—N1—C7	122.27 (11)	C14—C13—H13	123.0
C18—N2—C17	111.95 (11)	C15—C14—C13	123.06 (12)
C18—N2—C19	122.19 (11)	C15—C14—C18	107.76 (11)
C17—N2—C19	125.31 (11)	C13—C14—C18	129.16 (12)
C6—C1—C2	118.78 (13)	C14—C15—C16	122.69 (12)
C6—C1—C7	118.53 (11)	C14—C15—C17	108.64 (11)
C2—C1—C7	122.67 (12)	C16—C15—C17	128.56 (12)
C3—C2—C1	120.37 (13)	C10—C16—C15	114.41 (11)
C3—C2—H2	119.8	C10—C16—H16	122.8
C1—C2—H2	119.8	C15—C16—H16	122.8
C4—C3—C2	120.44 (13)	O3—C17—N2	126.28 (12)
C4—C3—H3	119.8	O3—C17—C15	128.21 (12)
C2—C3—H3	119.8	N2—C17—C15	105.50 (10)
C3—C4—C5	119.48 (13)	O4—C18—N2	125.85 (13)
C3—C4—H4	120.3	O4—C18—C14	128.01 (12)
C5—C4—H4	120.3	N2—C18—C14	106.14 (11)
C6—C5—C4	120.03 (13)	N2—C19—C21	111.00 (10)
C6—C5—H5	120.0	N2—C19—C20	109.47 (11)
C4—C5—H5	120.0	C21—C19—C20	115.67 (11)
C5—C6—C1	120.89 (13)	N2—C19—H19	106.7
C5—C6—H6	119.6	C21—C19—H19	106.7
C1—C6—H6	119.6	C20—C19—H19	106.7
N1—C7—C8	111.52 (11)	C19—C20—H20A	109.5
N1—C7—C1	109.15 (10)	C19—C20—H20B	109.5
C8—C7—C1	116.11 (11)	H20A—C20—H20B	109.5
N1—C7—H7	106.5	C19—C20—H20C	109.5
C8—C7—H7	106.5	H20A—C20—H20C	109.5
C1—C7—H7	106.5	H20B—C20—H20C	109.5
C7—C8—H8A	109.5	C26—C21—C22	118.67 (13)
C7—C8—H8B	109.5	C26—C21—C19	119.05 (12)
H8A—C8—H8B	109.5	C22—C21—C19	122.29 (12)
C7—C8—H8C	109.5	C23—C22—C21	120.35 (13)
H8A—C8—H8C	109.5	C23—C22—H22	119.8
H8B—C8—H8C	109.5	C21—C22—H22	119.8
O1—C9—N1	126.36 (13)	C24—C23—C22	120.37 (12)
O1—C9—C10	127.63 (12)	C24—C23—H23	119.8
N1—C9—C10	106.01 (11)	C22—C23—H23	119.8
C16—C10—C11	122.80 (12)	C23—C24—C25	119.57 (13)
C16—C10—C9	129.12 (12)	C23—C24—H24	120.2
C11—C10—C9	108.05 (11)	C25—C24—H24	120.2
C13—C11—C10	123.07 (12)	C26—C25—C24	120.09 (13)
C13—C11—C12	129.09 (12)	C26—C25—H25	120.0
C10—C11—C12	107.84 (11)	C24—C25—H25	120.0
O2—C12—N1	125.16 (13)	C25—C26—C21	120.93 (13)
O2—C12—C11	128.83 (13)	C25—C26—H26	119.5
N1—C12—C11	106.01 (10)	C21—C26—H26	119.5
			
C6—C1—C2—C3	−0.06 (19)	C13—C14—C15—C16	0.31 (19)
C7—C1—C2—C3	178.47 (13)	C18—C14—C15—C16	−177.85 (11)
C1—C2—C3—C4	−0.5 (2)	C13—C14—C15—C17	176.77 (11)
C2—C3—C4—C5	0.9 (2)	C18—C14—C15—C17	−1.39 (14)
C3—C4—C5—C6	−0.8 (2)	C11—C10—C16—C15	−0.13 (18)
C4—C5—C6—C1	0.2 (2)	C9—C10—C16—C15	177.67 (12)
C2—C1—C6—C5	0.21 (19)	C14—C15—C16—C10	0.26 (18)
C7—C1—C6—C5	−178.38 (12)	C17—C15—C16—C10	−175.45 (12)
C9—N1—C7—C8	66.11 (16)	C18—N2—C17—O3	177.94 (13)
C12—N1—C7—C8	−118.32 (13)	C19—N2—C17—O3	6.4 (2)
C9—N1—C7—C1	−63.51 (15)	C18—N2—C17—C15	−0.74 (14)
C12—N1—C7—C1	112.06 (13)	C19—N2—C17—C15	−172.31 (12)
C6—C1—C7—N1	−57.40 (14)	C14—C15—C17—O3	−177.32 (13)
C2—C1—C7—N1	124.07 (13)	C16—C15—C17—O3	−1.1 (2)
C6—C1—C7—C8	175.54 (12)	C14—C15—C17—N2	1.33 (13)
C2—C1—C7—C8	−2.99 (18)	C16—C15—C17—N2	177.52 (12)
C12—N1—C9—O1	−179.01 (13)	C17—N2—C18—O4	−179.55 (12)
C7—N1—C9—O1	−3.1 (2)	C19—N2—C18—O4	−7.7 (2)
C12—N1—C9—C10	0.25 (14)	C17—N2—C18—C14	−0.08 (14)
C7—N1—C9—C10	176.20 (11)	C19—N2—C18—C14	171.80 (11)
O1—C9—C10—C16	0.9 (2)	C15—C14—C18—O4	−179.61 (13)
N1—C9—C10—C16	−178.40 (13)	C13—C14—C18—O4	2.4 (2)
O1—C9—C10—C11	178.91 (13)	C15—C14—C18—N2	0.93 (14)
N1—C9—C10—C11	−0.33 (13)	C13—C14—C18—N2	−177.07 (12)
C16—C10—C11—C13	−0.58 (19)	C18—N2—C19—C21	120.96 (13)
C9—C10—C11—C13	−178.79 (12)	C17—N2—C19—C21	−68.29 (16)
C16—C10—C11—C12	178.50 (12)	C18—N2—C19—C20	−110.14 (13)
C9—C10—C11—C12	0.29 (13)	C17—N2—C19—C20	60.61 (16)
C9—N1—C12—O2	−179.90 (13)	N2—C19—C21—C26	−81.30 (15)
C7—N1—C12—O2	4.0 (2)	C20—C19—C21—C26	153.20 (13)
C9—N1—C12—C11	−0.07 (14)	N2—C19—C21—C22	99.25 (14)
C7—N1—C12—C11	−176.18 (11)	C20—C19—C21—C22	−26.25 (18)
C13—C11—C12—O2	−1.3 (2)	C26—C21—C22—C23	1.0 (2)
C10—C11—C12—O2	179.68 (13)	C19—C21—C22—C23	−179.51 (12)
C13—C11—C12—N1	178.86 (12)	C21—C22—C23—C24	0.3 (2)
C10—C11—C12—N1	−0.15 (13)	C22—C23—C24—C25	−1.2 (2)
C10—C11—C13—C14	1.07 (18)	C23—C24—C25—C26	0.9 (2)
C12—C11—C13—C14	−177.80 (12)	C24—C25—C26—C21	0.5 (2)
C11—C13—C14—C15	−0.94 (18)	C22—C21—C26—C25	−1.4 (2)
C11—C13—C14—C18	176.79 (12)	C19—C21—C26—C25	179.11 (13)

### Hydrogen-bond geometry (Å, º)

**Table:**

*D*—H···*A*	*D*—H	H···*A*	*D*···*A*	*D*—H···*A*
C6—H6···O1^i^	0.95	2.50	3.4129 (16)	162
C7—H7···O4^ii^	1.00	2.49	3.1840 (16)	126
C20—H20*C*···O1^iii^	0.98	2.56	3.4948 (17)	161
C23—H23···O1^iv^	0.95	2.57	3.4847 (18)	161

Symmetry codes: (i) *x*+1, *y*, *z*; (ii) −*x*+1, *y*+1/2, −*z*+2; (iii) −*x*, *y*−1/2, −*z*+1; (iv) −*x*−1, *y*−1/2, −*z*+1.
